# Bacterial, Clinical, and Antimicrobial Profile of Gram-Negative Community-Acquired Infections in a Rural Tertiary Care Hospital in India

**DOI:** 10.7759/cureus.79003

**Published:** 2025-02-14

**Authors:** Prathamesh P Dole, Satyajeet Pawar, Satish Patil

**Affiliations:** 1 Department of Microbiology, Krishna Institute of Medical Sciences, Krishna Vishwa Vidyapeeth, Karad, IND

**Keywords:** antimicrobial resistance, antimicrobial stewardship, community-acquired infections, gram-negative bacilli, multidrug-resistant pathogens

## Abstract

Background

Gram-negative bacilli, such as *Escherichia coli*, *Pseudomonas aeruginosa,* *Klebsiella *species (spp.), and *Proteus *species (spp.), are major causes of community-acquired infections (CAI). The rise in antibiotic use, especially in low- and middle-income countries, has led to increased antimicrobial resistance, with multidrug-resistant Gram-negative bacilli (MDR-GNB) becoming a global concern. This resistance contributes to longer hospital stays, increased mortality rates, and higher healthcare costs. The study aims to evaluate the bacterial, clinical, and antimicrobial profile of Gram-negative community-acquired infections (CAI) in a hospital setting to understand the prevalence better and inform strategies to tackle antibiotic resistance.

Material and methods

This study at Krishna Hospital and Medical Research Centre, Karad, focused on outpatient department (OPD) patients with suspected clinical infections. Non-repetitive Gram-negative bacilli isolates were included. After obtaining ethical approval and patient consent, clinical specimens were obtained aseptically first. The specimens were Gram-stained, cultured on various agars, and further confirmed. Species identification and testing for antimicrobial susceptibility were performed using the VITEK® 2 (bioMérieux, Marcy-l'Étoile, France) COMPACT Automated System to determine susceptibility profiles.

Result

A total of 518 specimens were processed, resulting in 102 Gram-negative bacilli isolates. The study revealed that community-acquired infections were most common in individuals aged 51-60 years (22, 21.57%). Men were more affected (62, 60.78%) than female patients (40, 39.22%). The most frequent pathogen was* Pseudomonas aeruginosa *(33, 32.35%), followed by *Escherichia coli* (29, 28.43%) and *Klebsiella *species (27, 26.47%). Discharge/pus was a frequent specimen source (66, 64.71%), followed by urine (30, 29.41%). These findings highlight age, gender, bacterial distribution, and the importance of pus and urine samples in diagnosis. The most sensitive drugs found in the study were fosfomycin (n = 54, 79.41%) and amikacin (n = 61, 67.03%); also, the most resistant drugs were cefuroxime (n = 43, 84.31%) and ciprofloxacin (n = 78, 76.47%).

Conclusion

Community-acquired infections represent a major public health challenge, shaped by factors such as age, gender, and pathogen diversity. These are more common in the 51-60 age group and show a male predominance due to behavioral and exposure factors in the study. Frequent pathogens include *Pseudomonas aeruginosa*, *Escherichia coli*, and *Klebsiella *spp., often involving skin and soft tissue infections (SSTI) and urinary tract infections (UTI). The study emphasizes the importance of understanding epidemiological trends, microbiological profiles, and trends of antibiotic resistance to improve treatment and prevention.

## Introduction

Most of the pathogenic Gram-negative bacilli belong to families such as Enterobacteriaceae and Pseudomonadaceae. These bacteria do not retain crystal violet dye during Gram staining, appearing rod-shaped and pink in color. Their cell envelopes are distinguished by a thin peptidoglycan cell wall positioned between their cytoplasmic cell membrane and a bacterial surface membrane [1]. They create a serious infection but also form a complication in treatment and healthcare systems [2]. Medically important Gram-negative bacilli causing community-acquired infection (CAI) include *Escherichia coli*, *Klebsiella *species (spp.), and *Pseudomonas *species (spp.).

A community-acquired infection is an infection outside the healthcare facility or an infection present at the time of hospital admission [3]. Infection carries significant morbidity and mortality throughout the area. For this, proper diagnosis and treatment in the early stages are very important. A recent survey shows that in low- and middle-income countries, the consumption rate of antibiotics has increased by 39% in the years 2000-2015. In India, the antibiotic consumption rate has increased by about 63% [4]. Due to the increase in the consumption of antibiotics, multidrug-resistant Gram-negative bacilli (MDR-GNB) organisms are prevailing more, which leads to an increase in resistance shown by the Gram-negative bacilli organisms.

Gram-negative bacilli can show resistance against one or more important groups of antibiotics. Multidrug-resistant Gram-negative bacilli are types of organisms that are resistant to an antimicrobial agent in three or more than three antimicrobial categories [5,6]. They can cause bacterial infections that lead to a serious and rapidly emerging threat to patients. According to a survey, the WHO said that worldwide, yearly deaths caused by multidrug-resistant organisms (MDRO) are 700,000, which is estimated to increase by about 10 million per year by 2050 [7].

Multidrug-resistant Gram-negative bacilli are frequently related to community-acquired infections, including urinary tract infections (UTI), respiratory infections such as community-acquired pneumonia (CAP), skin and soft tissue infections (SSTI), and gastrointestinal tract (GIT) infections [2]. Urinary tract infection (UTI), often caused by *Escherichia coli*, *Klebsiella pneumoniae*, and *Proteus mirabilis*, exhibits signs such as dysuria, urgency, frequency, and lower abdominal pain [8]. Community-acquired pneumonia, commonly due to *Klebsiella pneumoniae* and *Pseudomonas aeruginosa,* manifests as chest discomfort, fever, coughing, dyspnea, and sputum production [8]. Gastrointestinal infections are caused by pathogens such as *Salmonella *species, *Shigella *species, and pathogenic *Escherichia coli* strains that lead to diarrhea (watery or bloody), abdominal cramping, fever, nausea, and vomiting. Additionally, skin and soft tissue infections, particularly linked to *Pseudomonas aeruginosa*, may occur after water exposure and can result in cellulitis and wound infections. The prompt diagnosis of cases and appropriate antibiotic therapy are needed to manage these infections effectively.

Antibiotic overuse for community-acquired infections is frequently caused by self-medication, patient demands, and incorrect prescriptions for conditions. Many people abuse antibiotics due to misunderstandings about their efficacy, resulting in needless treatments and resistance. Healthcare providers may overprescribe antibiotics due to diagnostic uncertainty or time restrictions, and the availability of over-the-counter antibiotics in some locations contributes to misuse. In addition to raising healthcare expenses and accelerating antibiotic resistance, this usage has negative side effects such as disrupting the microbial ecosystem. Public education, more stringent laws, antibiotic stewardship initiatives, and easier access to diagnostic resources are all necessary to address this problem.

The overuse and misuse of antibiotics without a prescription from clinicians significantly contribute to the development of multidrug-resistant organisms (MDRO) [8]. These irresponsible practices allow bacteria to adapt and develop resistance, thereby restricting the use of existing antimicrobial treatments and rise in the global healthcare crisis. These multidrug-resistant organisms create a great threat to public health as limited treatment options available and a lack of newly developed antimicrobial medications. It has become of utmost concern in hospitals all around the world. Hence, the present study was carried out with the aim of evaluating the bacterial, clinical, and antimicrobial profiles of Gram-negative community-acquired infections in a rural tertiary care hospital in India.

## Materials and methods

Study design and sample size

This cross-sectional observational study was carried out in the Department of Microbiology of Krishna Institute of Medical Sciences, Krishna Vishwa Vidyapeeth, and Krishna Hospital and Medical Research Centre, Karad, from August 2022 to 2024. As per a study undertaken by Nagvekar et al. India, the study sample size was 89 [7]. The sample size calculation formula is as follows:

\[ n = \frac{Z^2 pq}{L^2} \]

where Z = confidence level of finding the sample (two), p = prevalence (33.33%), q = 100, p = 66.67%, and L = precision (10). With the above formula, the sample size for parameters from the study objectives with the above reference is as follows:

\[ n = \frac{4 \times 33.33 \times 66.67}{10^2} \]

\[ n = \frac{8888.44}{100} \]

n = 88.88 ≈ 89; to strengthen the study, the study included 102 isolates.

Inclusion criteria

Non-repetitive isolates of Gram-negative bacilli were taken from the clinical specimens. For this, the specimens were collected from outpatient department (OPD) patients of Krishna Hospital and Medical Research Centre, Karad, throughout the study period.

Collection of data

The study was conducted on outpatient department (OPD) patients with any suspected clinical infection at Krishna Hospital and Medical Research Centre, Karad. The data was collected from study participants using structured proforma. Details such as name, age, sex, OPD number, chief complaints (C/C), clinical data such as present and past medical history, antibiotics given, and details of clinical diagnosis were collected.

Sample collection

After approval by the Institutional Ethics Committee of Krishna Institute of Medical Sciences (protocol number: KIMSDU/IEC/02/2023), a total of 518 specimens were processed from all patients attending Krishna Hospital outpatient departments (OPD). The growth was obtained in 367 specimens. Among these, 102 isolates were identified as Gram-negative bacilli. Informed consent was taken from these patients. The clinical specimens such as pus, sputum, urine, and high vaginal swab from all groups of patients were included. Specimens were collected aseptically in sterile and appropriate containers and were processed as per standard procedure.

Microscopy

For the primary identification of Gram-negative bacilli based on their microscopic observation, a thin uniform smear was prepared. The heat-fixed smear was stained by the Gram stain technique and observed under the oil immersion objective of a light microscope. The smears were examined for the detection of Gram-negative bacilli. The size, shape, and arrangement of bacteria were noted.

Identification of Isolates

The isolates were recognized based on colony morphology on MacConkey agar, blood agar, and chocolate agar. Here, the Gram stain of the smear was made from the isolated colonies. VITEK® 2 (bioMérieux, Marcy-l'Étoile, France) GN 21341 card on the VITEK® 2 Compact Automated System was used for species-level identification of Gram-negative bacilli.

Antimicrobial Susceptibility Test

The antimicrobial sensitivity was done by using the VITEK® 2 Compact Automated System using VITEK® 2 AST-N405 and VITEK® 2 AST-N406.

Statistical analysis

The data obtained from this study was recorded using Microsoft Excel software (Microsoft Corporation, Redmond, WA). The analyzed results were presented using number (n) and percentage (%) values in a table, graphs, and a pie diagram. Statistical Package for Social Sciences (SPSS) version 28.0 (IBM Corp., Armonk, NY) was used to calculate the p-value using the chi-square test.

## Results

**Figure 1:**
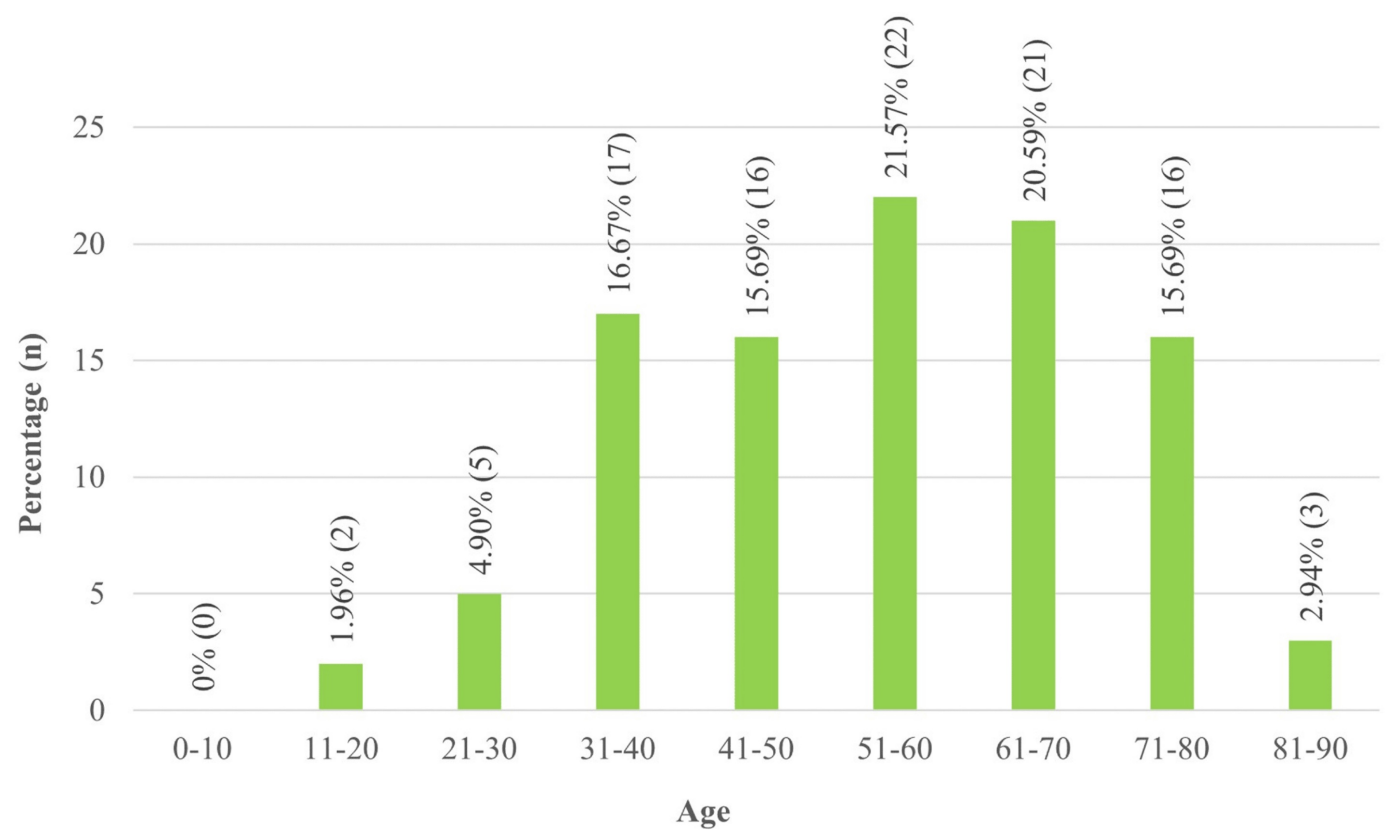
Age distribution of community-acquired infection %, percentage; n, number

A total of 518 specimens were processed, and growth was obtained in 367 specimens. Among these, 102 isolates were identified as Gram-negative bacilli. The prevalence of community-acquired infections differed according to age groups, with a minimal prevalence in teenagers aged 11-20 years (two, 1.96%). The highest prevalence is observed in the 51-60 age group, which is 22 (21.57%), followed closely by the 61-70 age group (21, 20.59%), as given in Figure 1. Infections declined among the elderly, with the 81-90 age group showing a low prevalence (three, 2.94%). These findings highlight a peak in middle-aged adults, with lower rates in younger and very elderly populations.

**Figure 2:**
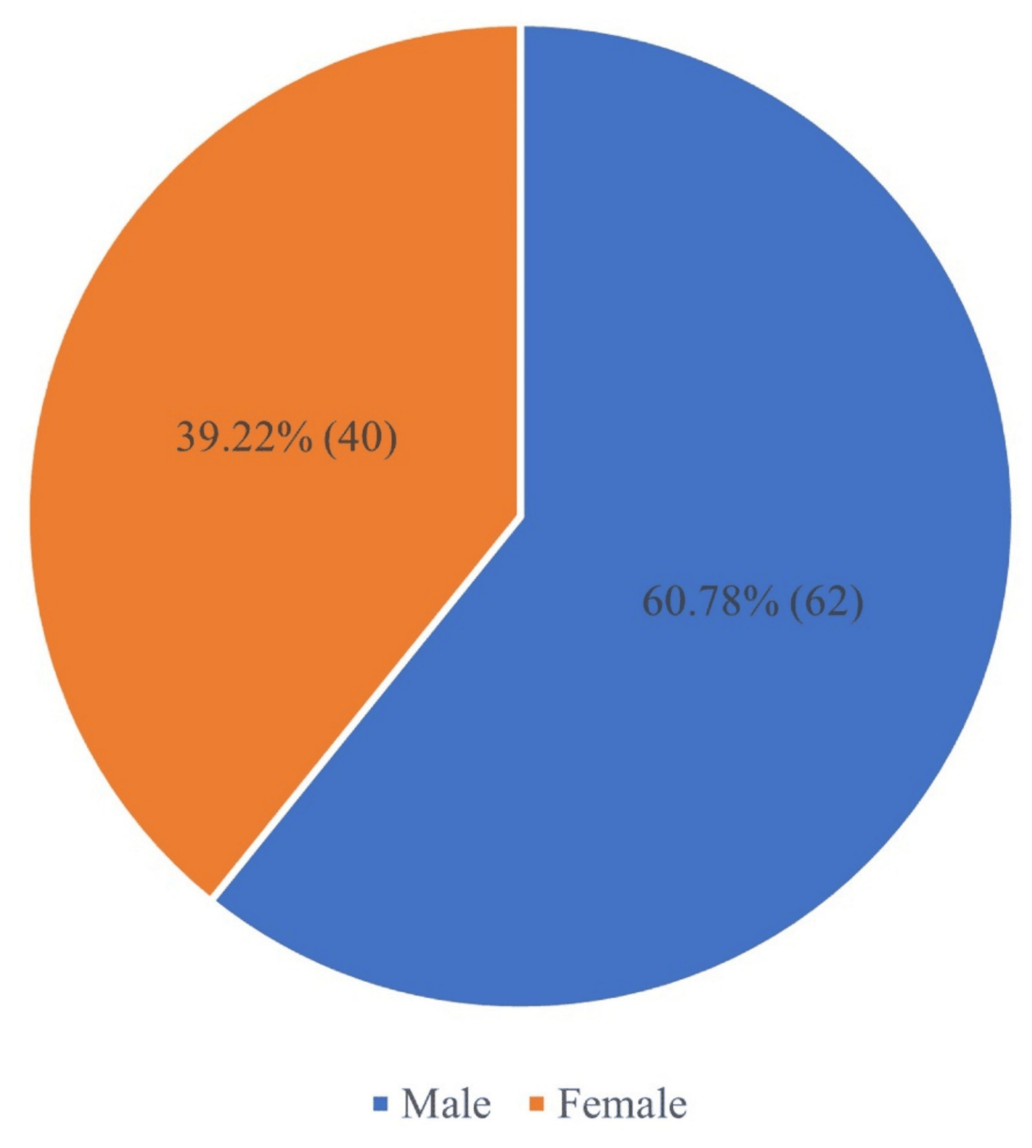
Gender-wise distribution of community-acquired infection %, percentage; n, number

The distribution of community-acquired infections shows a higher number of cases among men (62, 60.78%) and a lower number among women (40, 39.22%) as shown in Figure 2. Hence, the result shows that men are affected more than women in the observed population.

**Figure 3:**
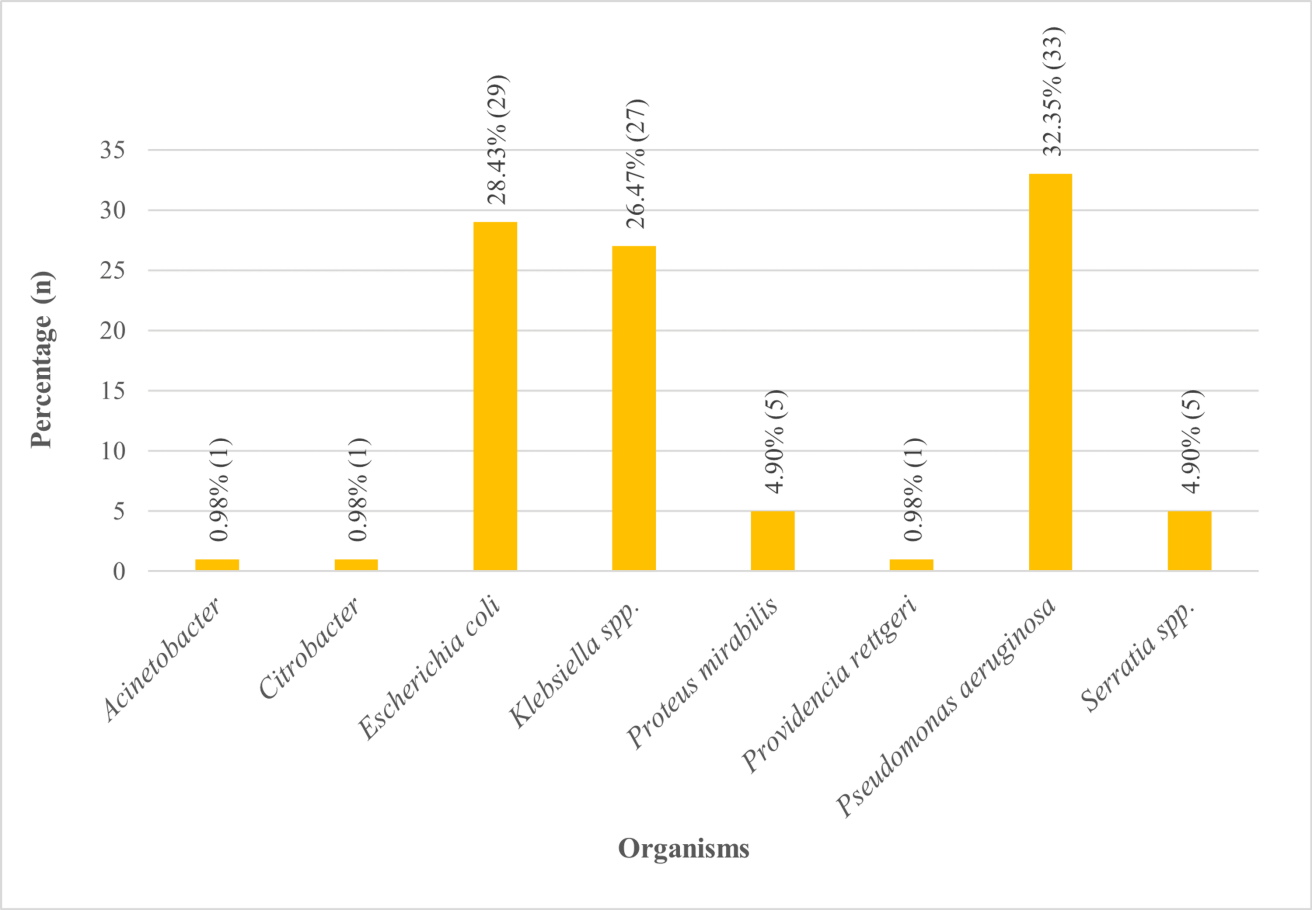
Organism-wise distribution of community-acquired infection %, percentage; n, number; spp., species

The distribution of community-acquired infections by organisms, as presented in Figure 3, highlights the prevalence of various bacterial species. Among the identified organisms, *Pseudomonas aeruginosa* was the most common, accounting for 33 (32.35%) cases. This was closely followed by *Escherichia coli*, which comprised 29 (28.43%), and *Klebsiella *spp., representing 27 (26.47%). Less commonly observed organisms included *Proteus mirabilis* and *Serratia *spp., each contributing five (4.90%). Rarely detected were *Acinetobacter*, *Citrobacter*, and *Providencia rettgeri*, each accounting for one (0.98%).

The distribution of community-acquired infections by specimen type as in Table 1 reveals the sources in which infections were identified. The most common specimen was discharge/pus, accounting for 66 (64.71%) cases, followed by urine, which comprised 30 (29.41%). Less frequently observed specimens included sputum, representing five specimens (4.90%), and high vaginal swab, which contributed one specimen (0.98%). The data highlights the predominance of pus and urine samples in diagnosing community-acquired infections, with other specimen types being less commonly involved.

**Table 1:** Specimen-wise distribution of community-acquired infection %: percentage

Number	Sample	Number (%)
1	Discharge/Pus	66 (64.71%)
2	Urine	30 (29.41%)
3	Sputum	5 (4.90%)
4	High Vaginal Swab	1 (0.98%)

**Figure 4:**
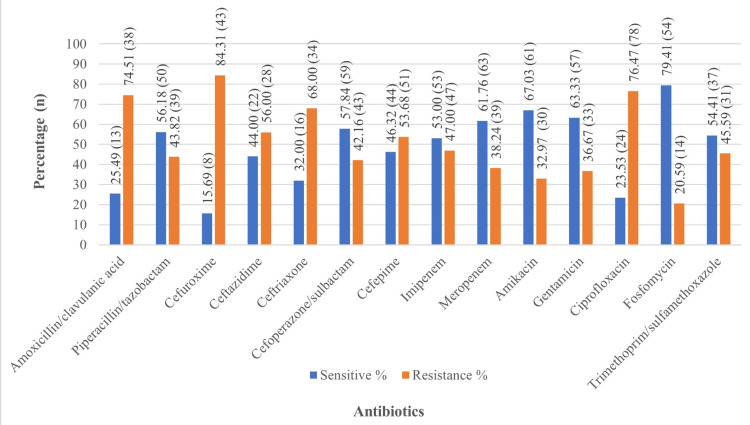
Antimicrobial susceptibility testing of community-acquired infection Chi-square test: 123.34; degree of freedom (df): 13; p < 0.0001, statistically significant n: number

Figure 4 shows that the sensitivity analysis highlights varied effectiveness among different antibiotic groups against community-acquired infections. Aminoglycosides such as amikacin and gentamicin showed high sensitivity rates (n = 61, 67.03%, and n = 57, 63.33%, respectively), demonstrating their effectiveness. Carbapenems, including meropenem (n = 63, 61.76%) and imipenem (n = 53, 53%), also exhibited good efficacy. Among β-lactam/β-lactamase inhibitors, fosfomycin showed the best sensitivity (n = 54, 79.41%), while piperacillin/tazobactam and cefoperazone/sulbactam showed moderate sensitivity (n = 50, 56.18%, and n = 59, 57.84%, respectively). However, cephalosporins such as cefuroxime and ceftriaxone displayed low sensitivity rates, highlighting reduced effectiveness within this group.

Resistance data underscores the challenges posed by certain antibiotic groups. Penicillin, represented by amoxicillin/clavulanic acid, exhibited high resistance (n = 38, 74.51%). Cephalosporins, including cefuroxime (n = 43, 84.31%) and ceftriaxone (n = 34, 68%), faced alarmingly high resistance rates. Fluoroquinolones, such as ciprofloxacin, showed resistance (n = 78, 76.47%), further limiting their utility. Conversely, aminoglycosides and carbapenems demonstrated relatively lower resistance rates, increasing their viability as choices for treatment.

## Discussion

Community-acquired infections (CAI) represent an important public health issue that impacts people of all ages and contributes to considerable morbidity and healthcare burden. Frequently, these diseases occur by a diverse range of bacterial pathogens and are influenced by factors such as age, gender, comorbid conditions, and environmental exposures. Understanding the epidemiological trends, microbiological profiles, and antibiotic susceptibility patterns of community-acquired infections is critical for effective management and prevention.

This study examined the prevalence of community-acquired infections across different age and gender groups, identified the predominant pathogens, and evaluated the specimen sources frequently related to these infections. Additionally, the antibiotic sensitivity and resistance patterns were analyzed to provide insights into the efficacy of commonly used antibiotics. The findings aim to inform clinical decision-making, guide empirical treatment strategies, and support public health efforts to combat antimicrobial resistance.

The study highlights significant age-related variability in the prevalence of community-acquired infections. It peaks in the 51-60 age group (22, 21.57%) and declines in older populations such as in 81-90 years old (three, 2.94%), likely due to reduced exposure. Low prevalence in the age group 11-20 (two, 1.96%) suggests robust immunity or lower exposure. The study by Todorovic Markovic et al. shows higher infections in the age groups 65-84 (n = 241, 51.2%) and 40-64 (n = 92, 19.5%) [3].

Non-fermenter organisms such as *Pseudomonas aeruginosa* (33,32.35%) and organisms from the family Enterobacteriaceae, that is, *Escherichia coli* (29, 28.43%) and *Klebsiella *spp. (27, 26.47%), were the most common pathogens found in this study. The organisms such as *Escherichia coli *and *Klebsiella *spp. were the most frequent pathogens in a similar study by Park et al. [9]. *Pseudomonas aeruginosa* is the most stubborn environmental bacterium, which is found in various burns and wound infections, commonly by Streeter and Katouli [10]. Additionally, a few studies indicate that *Escherichia coli* is the most significant pathogen causing UTI [3,11]. *Klebsiella pneumoniae* was found to be a cause of community-acquired pneumonia in the study by Bansal et al. [12]. ​​​​​​Less frequent organisms such as *Proteus mirabilis* and *Serratia *spp.,* *both* *five* *(4.90%), highlight a diverse microbiological profile. The study by Bandy et al. also gives a similar frequency of the isolates [13].

Men (62, 60.78%) were more impacted than women (40, 39.22%), possibly due to higher exposure or behavioral differences. The study by Bansal et al. shows that the number of men infected is more than the number of female patients [12].

Pus/discharge (66, 64.71%) and urine (30, 29.41%) dominate as specimen types, reflecting a focus on skin, soft tissue, and urinary tract infections. The common isolate found were *Pseudomonas aeruginosa*, *Klebsiella *spp., and *Escherichia coli*. Studies conducted by Osthoff et al. [11], Agnihotri et al. [14], and Rao et al. [15] on pus and wound infections show similar results.

The sensitivity analysis highlights the varying effectiveness of antibiotic groups against community-acquired infections, with aminoglycosides such as amikacin (n = 61, 67.03%) and gentamicin (n = 57, 63.33%) showing high sensitivity. Carbapenems such as meropenem (n = 63, 61.76%) and imipenem (n = 53, 53%) demonstrate good efficacy. The study by Kuinkel et al. reveals the higher sensitivity of gentamicin (68.4%) and lower sensitivity of imipenem, as well as meropenem (35%) [8]. Fosfomycin, a β-lactam/β-lactamase inhibitor, had the highest sensitivity (n = 54, 79.41%), while piperacillin/tazobactam (n = 50, 56.18%) and cefoperazone/sulbactam (n = 59, 57.84%) showed moderate effectiveness. In the study by Kuinkel et al., the sensitivity of piperacillin/tazobactam is lower (35%) and showed more resistance (53.5%) [8]. Conversely, cephalosporins (e.g., cefuroxime and ceftriaxone) exhibited low sensitivity, indicating reduced utility. High resistance rates in penicillin derivatives, cephalosporins, and fluoroquinolones highlight their reduced effectiveness, while aminoglycosides and carbapenems remain viable options. These findings emphasize the need for sensitivity-guided antibiotic selection and robust antimicrobial stewardship (AMS) to curb resistance and preserve treatment efficacy.

Limitations

One of the limitations of this study is the relatively small sample size, which may limit the statistical power and validity of the findings. In addition, the specimens were sourced from a single hospital, which may limit the applicability of the study to different populations or healthcare settings.

## Conclusions

This study highlights the public health burden of community-acquired infections (CAI) and the need for targeted interventions. Community-acquired infections were most common in the 51-60 age group and showed a male predominance due to behavioral and exposure factors. Frequent pathogens include *Pseudomonas aeruginosa*, *Escherichia coli*, and *Klebsiella *spp., with infections often involving the skin, soft tissue, and urinary tract. Drugs such as aminoglycosides and carbapenems remain effective, while cephalosporins and fluoroquinolones show high resistance, stressing the importance of sensitivity-guided antibiotic use. The findings emphasize the need for robust surveillance, preventive measures, mandatory screening protocols, and integration into community-level antimicrobial stewardship (AMS) programs to combat resistance and improve infection management.
